# Bilateral bullous keratopathy secondary to melting keratitis in a Suri alpaca (*Vicugna pacos*)

**DOI:** 10.1002/ccr3.1389

**Published:** 2018-02-15

**Authors:** Alexandre Guyonnet, Aurélie Bourguet, Elise Donzel, Guillemette Bataille, Quentin Pascal, Eve Laloy, Henri‐Jean Boulouis, Yves Milleman, Sabine Chahory

**Affiliations:** ^1^ CHUVA, Unité d'Opthalmologie Ecole Nationale Vétérinaire d'Alfort Université Paris ‐ Est Maisons‐Alfort F‐94700 France; ^2^ Unité de Pathologie des Animaux de Production Ecole Nationale Vétérinaire d'Alfort Université Paris ‐ Est Maisons‐Alfort F‐94700 France; ^3^ BioPôle, Unité d'Histologie et Anatomie pathologique Ecole Nationale Vétérinaire d'Alfort Université Paris ‐ Est Maisons‐Alfort F‐94700 France; ^4^ BioPôle, Unité de Bactériologie Ecole Nationale Vétérinaire d'Alfort Université Paris ‐ Est Maisons‐Alfort F‐94700 France

**Keywords:** Alpaca, bacterial keratitis, bullous keratopathy, cornea, edema, eye

## Abstract

An young alpaca was evaluated for bilateral progressive melting corneal ulcers and developped secondary bullous keratopathy during hospitalization. The tragic progression of melting ulcers in both eyes observed in our case leads us to recommend a rapid intensive medical therapy in young and debilitated alpacas presenting a corneal ulcer.

## Introduction

Corneal disease is the most common ocular abnormality seen in New World camelids 1. Probably traumatic corneal ulcers are the most frequently seen corneal disease, but other conditions were described including laceration, foreign bodies, stromal abscesses, calcific band keratopathy and epithelial inclusion cyst 2, 3, 4.

Bullous keratopathy has been documented in humans and dogs with various ocular diseases 5, 6. To date, bullous keratopathy has not been reported to occur in camelids.

The purpose of this report is to describe clinical and histologic features of bilateral bullous keratopathy secondary to melting keratitis in a three‐month‐old alpaca.

## Case Report

A three‐month‐old sexually intact female Suri alpaca was presented for evaluation of delayed growth, overall poor body condition and a two‐week history of lack of appetite. The initial physical examination revealed dull hair coat, thin body condition (2/9). Serum chemistry and urinalysis showed no significant abnormalities other than mild hypoproteinemia (total serum protein, 53 g/L; reference range 55–70 g/L). Serial fecal flotations and bovine herpesvirus Type 1 serology gave negative results. Ultrasound of the abdomen failed to show any abnormality. Based on the reported feeding regimen, a presumptive diagnosis of protein‐energy malnutrition was made and a change in diet with high‐energy density food was prescribed.

Three days after admission, extensive superficial corneal ulcerations in both eyes associated with mild corneal edema were observed despite no history of ocular trauma. Medications included topical neomycin‐polymyxin B (Tevemyxine ophthalmic ointment, TVM, Lempdes, France) every 8 h in both eyes. On the second day of treatment, bilateral marked complication of melting keratitis was noticed with the presence of malacic corneal material in the ulcer area. The topical therapy was changed to a fortified solution of gentamicin 13.6 mg/mL every 4 h (Forticine, Vetoquinol, Paris, France). Subcutaneous flunixin meglumine 1 mg/kg (Finadyne, Intervet, Beaucouze, France) every 24 h was added.

**Figure 1:**
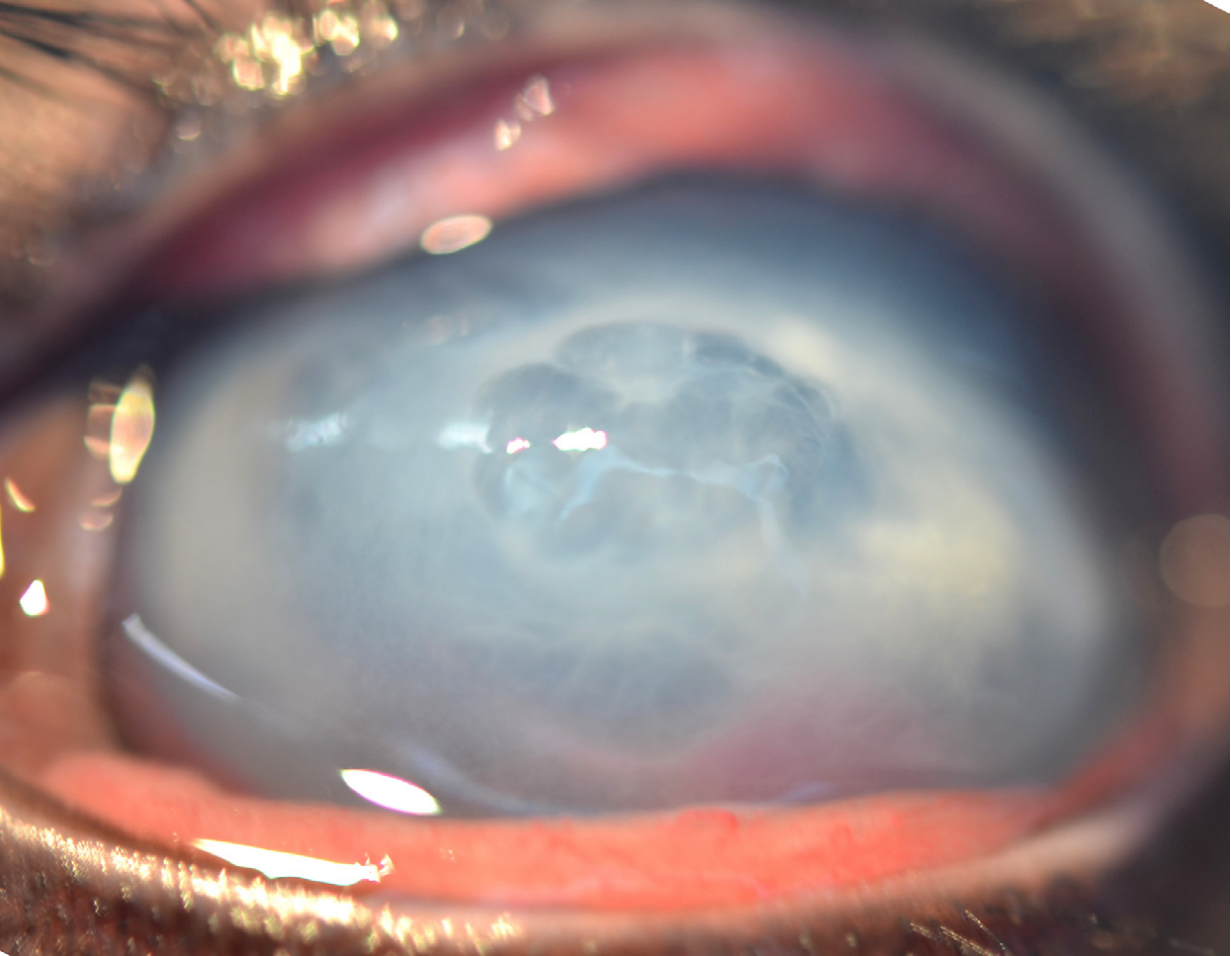
Photograph from the left eye at the first examination. Note the large stromal bullae, diffuse corneal edema and multifocal areas of stromal infiltrate.

Three days later, a complete ocular examination, performed by an ophthalmologist, revealed an absent menace response in both eyes but preserved palpebral and dazzle reflexes. Pupillary light reflexes were not observable due to corneal opacification. A marked blepharospasm with purulent discharge was noted bilaterally. Slit‐lamp biomicroscopy examination (SL‐15 Portable Slit lamp, Kowa Company, Tokyo, Japan) revealed marked conjunctival hyperemia and marked diffuse stromal edema with a central 8‐mm‐large bulla, involving the anterior half of the corneal stroma in both eyes (Fig. 1). The surrounding cornea appeared malacic with multifocal yellow areas of stromal infiltrates. Fluorescein staining was positive on the bullae. Intraocular pressure was not measured due to the apparent corneal fragility in both eyes. The anterior segment was difficult to assess but mild hypopyon was present in the left eye. Funduscopic examination in either eye could not be performed because of severe corneal opacification.

The most likely diagnosis was bullous keratopathy secondary to infectious ulcerative keratitis. The differential diagnosis, including other causes of bullous keratopathy, such as endothelial dystrophy, anterior uveitis or endotheliitis, appeared less likely due to the initial presence of corneal ulcers.

Corneal cytology revealed clumps of mucous material with a moderate number of neutrophils, few cytologically normal corneal epithelial cells and no visible bacteria or fungal organisms.

Options for therapy were discussed and included intensive medical treatment with close monitoring, keratoplasty or conjunctival graft. A topical intensive medical therapy was decided by the owner: fortified solution of gentamicin q2h OU (Forticine, Vetoquinol, Paris, France), heterologous serum q2h OU, 1% atropine q8h OU (VT Doses Atropine 1%, TVM, Lempdes, France) and 0.1% dimeticone q8h OU (Ophtasiloxane, Alcon, Reuil Malmaison, France), a tear‐substitute with osmotic power. Systemic flunixin meglumine (Finadyne, Intervet, Beaucouze, France) was continued.

After 3 days of this treatment, the patient was unable to rise and eat by itself and developed cutaneous myiasis secondary to decubitus ulcer. Despite a mild regression of the corneal edema, the stromal bullae remained unchanged. Culture yielded *Staphylococcus haemolyticus,* resistant to all antibiotics tested except polymyxin B (Table 1). Fungal culture was negative.

**Table 1:** Antimicrobial susceptibility test results for the *Staphylococcus haemolyticus* isolate

Name of chemicals	Susceptibility
Penicillin	R
Amoxicillin	R
Amoxicillin–clavulanate	R
Cefoxitin	R
Cephalexin	R
Ceftiofur	R
Erythromycin	R
Doxycycline	R
Enrofloxacin	R
Marbofloxacin	R
Kanamycin	R
Tobramycin	R
Gentamicin	R
Lincomycin	R
Polymyxin B	S

S, Susceptible; R, Resistant.

**Figure 2:**
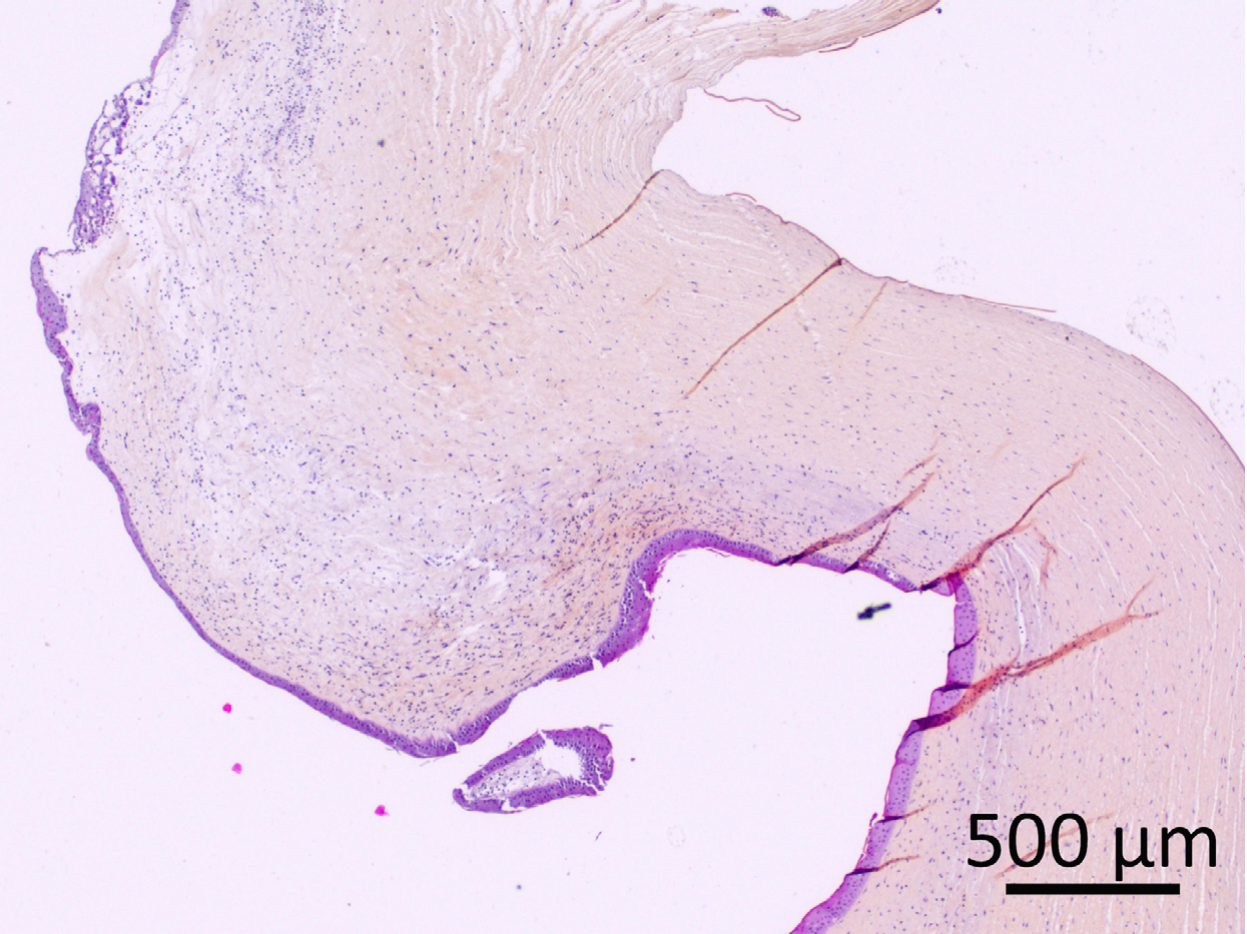
Cornea of the right eye: Focally extensive marked corneal edema, moderate to marked suppurative keratitis and limbal neovascularization. HES stain; bar = 500 *μ*m.

**Figure 3:**
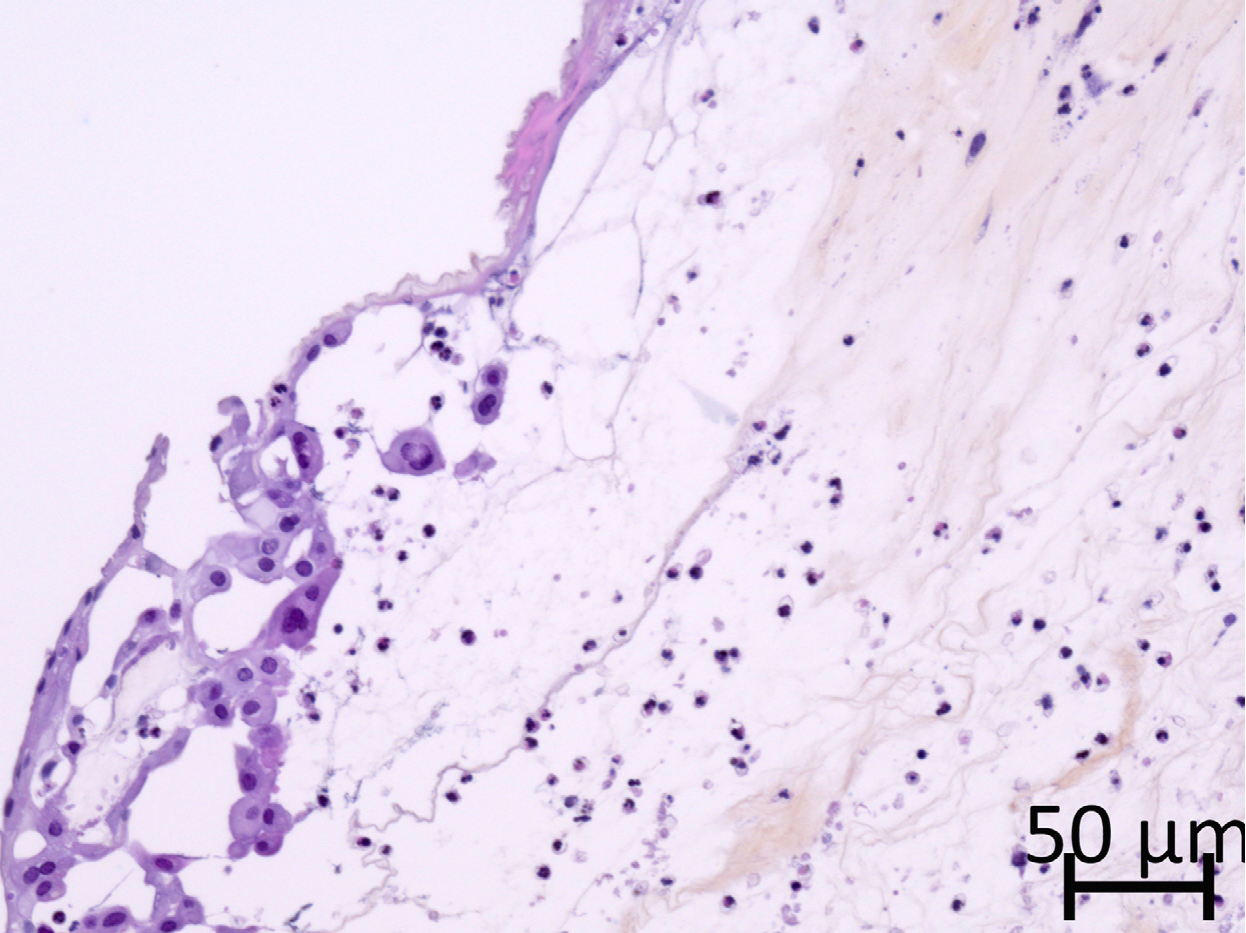
Cornea of the right eye: ulcerative keratitis with fibrin exudation, rare multinucleated epithelial cells and stromal edema. HES stain; bar = 50 *μ*m.

In view of the deterioration of the patient's general condition, the owner elected euthanasia with postmortem examination. No evidence of systemic disease was found at necropsy, and the presumptive clinical diagnosis of primary protein malnutrition was confirmed. Microscopic ocular changes included marked bilateral corneal edema (Fig. 2), associated with moderate ulcerative and suppurative keratitis with rare multinucleated epithelial cells (Fig. 3), limbal neovascularization and mild neutrophilic anterior uveitis with hypopyon. No bacteria or fungal organisms were found.

## Discussion

Corneal disease is the most frequently ocular abnormality recorded in camelids 1. Among llamas presented to the Colorado State veterinary teaching hospital for eye diseases, 41% had active corneal disease, of which more than half were ulcers 2. Camelids have large prominent eyes, which may predispose them to traumatic corneal ulcers or lacerations. Corneal trauma may be caused by fighting, penetration of the cornea with plant material, or prolonged recumbency 2. In a retrospective study of sick neonatal New World Camelids, 21% of cases examined were found to suffer from an ocular problem, with ulcerative keratitis as the most frequent lesion (67%) 7. In this group of crias, altered mentation resulting in recumbency could have increased the likelihood of sustained environmental ocular trauma.

In our case, the young alpaca presented bilateral corneal ulcerations over a short period after its admission. The origin of the corneal disease remains unknown but it may be secondary to trauma or exposure keratitis associated with poor general health. Microscopic examination of the cornea in this case revealed sparse multinucleated cells suggestive of syncytial cells that may be compatible with a viral infection. Recent anecdotal reports have implicated a herpesvirus as the cause of superficial dendritic ulcers in camelids 1. These ulcers display features similar to those seen in cats infected by herpesvirus type 1. Although the organism was not positively detected, the good outcome of corneal ulcers following antiviral therapy suggested a viral origin 1. In this report, the implication of a herpesvirus cannot be confirmed in the absence of paraffin‐fixed tissue for PCR but seems unlikely in the absence of dendritic ulcers at the initial presentation.

Melting corneal ulcers are a complication of corneal ulcers due to an excess of proteinases 8. They occur when there is an imbalance between proteinases and proteinase inhibitors in favor of the proteinases, which leads to pathologic degradation of the stromal collagen and proteoglycans in the cornea 9, 10. Microbial infection is usually suspected to drive the inflammatory condition responsible for the melting process although this cannot always be demonstrated 5. Aggressive treatment with topical antimicrobials to battle a potential infection and with anticollagenases to directly counter collagenolysis is indicated to stop progression of the melting process 5, 11. The results of medical treatment are considered to be variable 12. Several factors including the advance state of the disease in veterinary patients, the inability of owners to adequately follow drug prescriptions, drug resistance of microbial pathogens and problems with patient compliance can negatively influence the course of the disease 13. In a significant number of cases, corneal melting leads to progressive ulceration and even perforation, necessitating globe stabilizing or enucleation. In this extreme case, the owner feel forced to elect euthanasia over surgical treatment.

In this report, corneal culture identified *Staphylococcus haemolyticus*. This bacteria is a progressively emerging threat in the area of infectious agents regarding of carriage of various antibiotic resistance genes 14. Although *mec* genes were not sought, the profile of antibiotic susceptibility of the strain identified in this report is analogous to the profile of methicillin‐resistant *Staphylococcus spp* (MRS). The route of bacterial transmission is undetermined in the case presented here but may have been acquired from multiple potential sources, such as MRS‐positive veterinary staff, veterinary clinic environment or have been selected by prior administration of antimicrobials 15. Resistance to a large number of commonly used antibiotics and pathogenicity of this bacteria could explain the rapid evolution of melting process despite intensive medical therapy. In this case, the pathogen was susceptible to only polymixin B but a progression of the melting process was observed despite the use of this antibiotic. The most likely reason for this discrepancy is the initial low instillation frequency, which did not allow the sterilization of the corneal ulcer.

In the absence of bacteria visible on cytologic and histopathologic examination, the possibility of sample contamination or isolation of incidental microflora cannot be ruled out. Although this hypothesis is unlikely as the sample for culture was collected directly from corneal lesions, care was taken to avoid contact with other extraocular tissues during sample collection, the clinical findings were consistent with bacterial keratitis and samples were immediately inserted into microbiologic transport medium and processed for culture without lengthy delays.

In this report, the main sign of corneal disease was the acute onset of a bilateral bullous keratopathy. In humans and dogs, this condition is known as a complication of corneal edema in melting keratitis, among other causes 5, 6. In these cases, corneal edema is often secondary to anterior uveitis associated with ulceration. The formation of large stromal bullae is not a frequent clinical finding of bullous keratopathy in other species. However, New World camelids have a described propensity to develop marked corneal edema following trauma, uveitis and surgery 1. A study showed that the number of endothelial cells in camelids was only slightly lower than in other species but frequent variability in cell size and shape of the endothelial cells of normal camelids was reported 16. These are common findings in other species having pathologically decreased endothelial cell density, which suggests that clinically normal alpacas and llamas have a potential increased vulnerability to the development of corneal edema. In this case, moderate anterior uveitis associated to melting keratitis and inherently increased endothelial vulnerability may have exacerbated the development of corneal edema and the formation of large stromal bullae.

This case describes the clinical and histopathological features of a bilateral bullous keratopathy as a complication of melting ulcers in an alpaca. In this report, the initial cause of the ulcers remains unknown but it may be secondary to trauma or exposure keratitis due to poor general health. The tragic progression in both eyes observed in our case leads us to recommend a rapid intensive medical therapy in young and debilitated alpacas presenting a corneal ulcer.

## Authorship

AG, AB, ED and SC: designed the concept of the clinical case report and performed the clinical examination, and follow‐up of the patient, GB and YM: performed the complementary examinations and treatment, YJB: performed the culture and sensibility testings, QP and EV: performed the pathological procedures. All authors contributed with the writing and approval of the final manuscript.

## Conflict of Interest

None declared.
